# The Chloroplast Envelope Protease FTSH11 – Interaction With CPN60 and Identification of Potential Substrates

**DOI:** 10.3389/fpls.2019.00428

**Published:** 2019-04-05

**Authors:** Zach Adam, Elinor Aviv-Sharon, Alona Keren-Paz, Leah Naveh, Mor Rozenberg, Alon Savidor, Junping Chen

**Affiliations:** ^1^The Robert H. Smith Institute of Plant Sciences and Genetics in Agriculture, The Hebrew University of Jerusalem, Rehovot, Israel; ^2^de Botton Institute for Protein Profiling, The Nancy and Stephen Grand Israel National Center for Personalized Medicine, Weizmann Institute of Science, Rehovot, Israel; ^3^Plant Stress and Germplasm Development Unit, USDA-ARS, Lubbock, TX, United States

**Keywords:** FTSH11, chloroplast, envelope, proteolysis, AAA protease, CPN60

## Abstract

FTSH proteases are membrane-bound, ATP-dependent metalloproteases found in bacteria, mitochondria and chloroplasts. The product of one of the 12 genes encoding FTSH proteases in Arabidopsis, FTSH11, has been previously shown to be essential for acquired thermotolerance. However, the substrates of this protease, as well as the mechanism linking it to thermotolerance are largely unknown. To get insight into these, the FTSH11 knockout mutant was complemented with proteolytically active or inactive variants of this protease, tagged with HA-tag, under the control of the native promoter. Using these plants in thermotolerance assay demonstrated that the proteolytic activity, and not only the ATPase one, is essential for conferring thermotolerance. Immunoblot analyses of leaf extracts, isolated organelles and sub-fractionated chloroplast membranes localized FTSH11 mostly to chloroplast envelopes. Affinity purification followed by mass spectrometry analysis revealed interaction between FTSH11 and different components of the CPN60 chaperonin. In affinity enrichment assays, CPN60s as well as a number of envelope, stroma and thylakoid proteins were found associated with proteolytically inactive FTSH11. Comparative proteomic analysis of WT and knockout plants, grown at 20°C or exposed to 30°C for 6 h, revealed a plethora of upregulated chloroplast proteins in the knockout, some of them might be candidate substrates. Among these stood out TIC40, which was stabilized in the knockout line after recovery from heat stress, and three proteins that were found trapped in the affinity enrichment assay: the nucleotide antiporter PAPST2, the fatty acid binding protein FAP1 and the chaperone HSP70. The consistent behavior of these four proteins in different assays suggest that they are potential FTSH11 substrates.

## Introduction

FTSH proteases are membrane-bound, ATP-dependent metalloproteases, initially identified in *Escherichia coli* (Tomoyasu et al., 1993), and later in all prokaryotes and organelles of prokaryotic origin in eukaryotic cells (Schnall et al., 1994; Lindahl et al., 1996; Sakamoto et al., 2002; Adam et al., 2005; Ito and Akiyama, 2005; Langklotz et al., 2012). FTSH proteases are anchored to cellular membranes by one or two *trans*-membrane helices (TMs) and form homo- or hetero-hexameric complexes. X-ray crystallography of the cytosolic region of bacterial FTSHs revealed that their active sites are found within a cage-like structure, secluded from the cellular environment (Krzywda et al., 2002; Bieniossek et al., 2006, 2009; Suno et al., 2006), qualifying them as self-compartmentalizing proteases (Lupas et al., 1997). Like all other ATP-dependent proteases, the ATPase function is essential for substrate recognition, its unfolding and translocation to the proteolytic sites (Ito and Akiyama, 2005; Langklotz et al., 2012). Thus, the proteolytic activity of FTSHs is dependent on their ATPase one, but the ATPase can confer chaperone activity that is independent of the proteolytic one. In microbial systems, the usually single FtsH gene-product is essential to their survival at elevated temperatures and is also involved in protection against other environmental stresses (Deuerling et al., 1995; Tomoyasu et al., 1995; Beier et al., 1997; Bourdineaud et al., 2003). It functions as a molecular chaperone and protease and helps organisms to maintain cell homeostasis under optimal and stress conditions (Schumann, 1999; Ito and Akiyama, 2005; Langklotz et al., 2012).

In plants, FTSH proteases are encoded by multiple genes, 12 in the case of Arabidopsis (Adam et al., 2005; Wagner et al., 2012). The best-characterized plant FTSH is that of the hetero-complex found in the thylakoid membrane. It is composed of two types of subunits, A (FTSH1 and FTSH5) and B (FTSH2 and FTSH8). The proteins within a type are redundant, but the presence of both types is essential for the accumulation of a stable complex (Yu et al., 2004; Zaltsman et al., 2005; Adam et al., 2006; Sakamoto, 2006). This complex, designated FTSH1-2-5-8, as well as its homologs in cyanobacteria and algae, is mainly involved in repairing the photosynthetic machinery from damages associated with high-light stress in the context of photoinhibition (Lindahl et al., 2000; Bailey et al., 2002; Kato et al., 2012; Komenda et al., 2012; Malnoe et al., 2014), and also those incurred by heat stress (Yoshioka et al., 2006; Yamamoto et al., 2008). In addition, plant genomes encode proteolytically inactive homologs (FTSHi) (Sokolenko et al., 2002; Wagner et al., 2012), which have their ATPase domain intact but their proteolytic site is mutated. They were suggested to be involved in chloroplast development (Kadirjan-Kalbach et al., 2012; Lu et al., 2014), and recently it was demonstrated that they serve as a motor for protein import into chloroplasts (Kikuchi et al., 2018).

Unlike dozens of publications on the thylakoid FTSH1-2-5-8 complex and its physiological roles, reports on FTSH11 are scarce. FTSH11 was first characterized in Arabidopsis mitochondria, forming a complex together with FTSH4, homologous to the well-known *i*-AAA protease in yeast mitochondria (Urantowka et al., 2005). In that work it was localized to chloroplasts as well. Further confirmation and chloroplast envelope localization were obtained by MS and immunoblot analyses (Knopf et al., 2012; Wagner et al., 2016), but in the latter report FTSH11 could not be detected in purified mitochondria, leaving the question of dual targeting to both organelles ambiguous. Using a genetic approach, an Arabidopsis mutant that was sensitive to moderate heat stress (30°C) and defective in acquired thermotolerance was isolated, and the mutation was mapped to the gene encoding the FTSH11 protease (Chen et al., 2006). Unlike FTSH2 and FTSH5 mutants that are insensitive to heat, the FTSH11 mutant demonstrated reduced photosynthetic activity at elevated temperature (Chen et al., 2018), suggesting that different FTSH proteases have their own roles in response of plants to different stresses.

In the current study, we sought to determine whether the proteolytic or chaperone activities of FTSH11 were responsible for thermotolerance, to revisit the issue of its cellular location, to identify its potential partners and substrates, and to evaluate the proteomic consequences of its loss. We demonstrate here that the proteolytic activity of FTSH11 is indeed essential for growth at elevated temperatures; it is mostly located in chloroplasts; it interacts with the stromal chaperonin CPN60; and we identify a number of potential chloroplast substrates.

## Results

### The Proteolytic Activity of FTSH11 Is Essential for Thermotolerance

**FIGURE 1:**
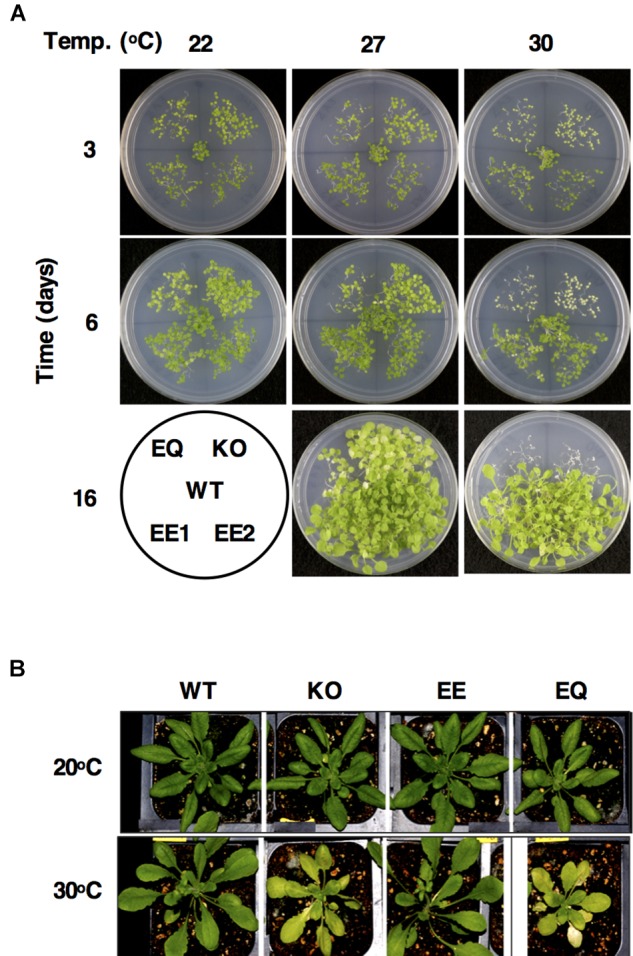
The proteolytic activity of FTSH11 is essential for thermotolerance. **(A)** Different Arabidopsis lines were grown on agar plates for 3, 6, or 16 days at 22, 27, or 30°C. The location of each line on the plate is indicated on the map at the lower left corner of the figure. WT, wild type; KO, FTSH11 knockout; EE1 and 2 – two different lines of FTSH11-HA (active protease); EQ – proteolytically inactive protease. **(B)** The same lines grown on soil at either 20 or 30°C.

In their original work on FTSH11, Chen et al. (2006) have shown that this protein is essential for survival of Arabidopsis seedlings at elevated temperatures. As the proteolytic activity of FTSH proteases is dependent on their ATPase domain for substrate recognition and unfolding, it raised the question whether thermotolerance was dependent on the proteolytic activity of FTSH, or else, the chaperone-like activity of this domain was sufficient for this function. To answer this question, two different transgenic lines were generated. The first line, designated EE, was FTSH11-knockout mutant, complemented with FTSH11 full-length cDNA, encoding also a HA tag at the C-terminus of the protein, under the control of the endogenous promoter. The second line, designated EQ, was an identical one, with the exception that it contained amino acid substitutions in the proteolytic site. His 620 and Glu621 that are part of the conserved Zn^2+^-binding site H-E-X-X-H were replaced by Gln. Such mutations are well documented to abolish the proteolytic activity of FTSH proteins in either *E. coli* or yeast mitochondria without affecting their ATPase activity (e.g., Karata et al., 1999; Leonhard et al., 1999). Seedlings of these lines, along with the WT and the FTSH11 knockout lines, were grown on plates at either 22, 27 or 30°C for 3, 6, or 16 days. As shown in Figure 1A, all lines grew at 22 and even at 27°C. At 30°C, the knockout lines complemented with the full-length cDNA of FTSH11 grew like WT, reconfirming that the loss of FTSH11 was sufficient to cause thermosensitivity. Moreover, this complementation also confirmed that tagging the protein with the HA tag did not interfere with its physiological activity. Nevertheless, a single amino acid substitution in the proteolytic domain of FTSH11 resulted in a thermosensitive phenotype, similar to the phenotype of the knockout line. Slower growth and a paler phenotype of these lines could be observed even at 22 and 27°C. When mature plants were subjected to heat for 10 days, similar behavior of the different genotypes was observed (Figure 1B). These results suggested that the proteolytic activity of FTSH11, and not only its ATPase one, is essential for tolerating elevated temperatures.

### FTSH11 Is Located in the Chloroplast Envelope

**FIGURE 2:**
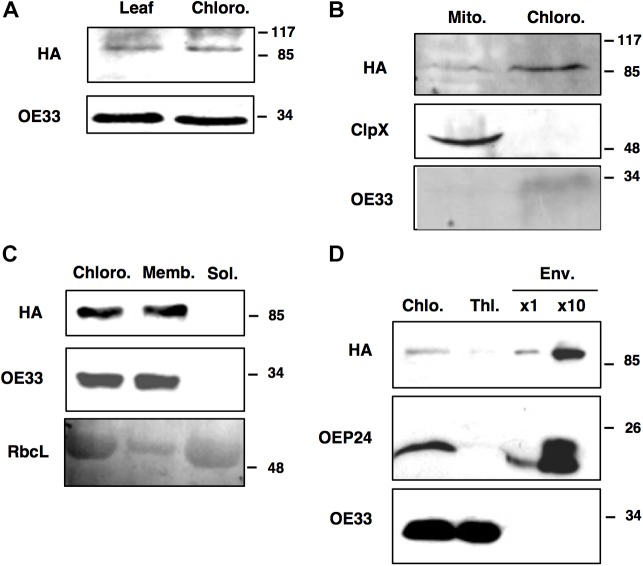
FTSH11 is located in the chloroplast envelope. Different extracts, organelles and fractions, from transgenic plants expressing FTSH11-HA, were subjected to imunoblot analysis with HA and control antibodies. **(A)** Total leaf and isolated chloroplast extracts. **(B)** Isolated mitochondria and chloroplasts. **(C)** Chloroplasts fractionated to membrane and soluble fractions. **(D)** Thylakoid and envelope membranes isolated on the same sucrose gradient. Marker proteins for the different compartments are: OE33 for thylakoids, ClpX for mitochondria, RbcL for stroma and OEP24 for envelopes. Antibodies used are indicated on the left and migration of MW markers (in kDa) are indicated on the right of each blot.

Prediction servers such as TargetP suggest that FTSH11 is located in chloroplasts. Using N-terminal signal sequences fused to GFP, Sakamoto and co-workers have concluded that FTSH11 is indeed targeted to chloroplasts (Sakamoto et al., 2003). Another study, making use of immunoblot analysis, suggested that FTSH11 is dually targeted to both chloroplasts and mitochondria (Urantowka et al., 2005). In a mass spectrometry (MS) analysis of chloroplast envelopes, in the context of a study on rhomboid proteases, FTSH11 was identified among 180 other proteins (Knopf et al., 2012). In line with the required multiple lines of evidence to define the subcellular location of plant proteins (Millar et al., 2009), we chose to use the transgenic plants expressing HA-tagged FTSH11 in a knockout background (the aforementioned EE plants) for cellular localization of this protein. This approach eliminated potential cross-reactivity associated with the use of antibodies against proteins belonging to gene families such as FTSH. Comparison of total leaf and chloroplast extracts, in immunoblot analysis with antibodies against the HA tag, loaded on the base of equal chlorophyll, revealed equal signals (Figure 2A), suggesting that the signal in the total extract was of chloroplast origin. When equal amounts of protein, extracted from isolated chloroplasts and mitochondria, were compared, chloroplasts displayed a much stronger HA signal (Figure 2B). Together, these results suggested that most, if not all of FTSH11 is located in chloroplasts.

When chloroplasts were sub-fractionated to membrane and soluble fractions, all the HA signal was associated with chloroplast membranes (Figure 2C). To distinguish between thylakoid and envelope membranes localization, the chloroplast membrane fraction was resolved by density gradient centrifugation on a sucrose gradient. The thylakoid fraction appeared to be somewhat contaminated with envelope proteins, as judged by the faint band of the envelope protein OEP24 in this fraction (Figure 2D). However, no trances of the thylakoid protein OE33 were found in the envelope fraction. As for the HA signal, it was associated with the envelope fraction, suggesting that FtsH11 is indeed located in the chloroplast envelope, in agreement with its identification in MS data [e.g., (Knopf et al., 2012; Simm et al., 2013)].

### FTSH11 Interacts With CPN60 and Other Chloroplast Proteins

**FIGURE 3:**
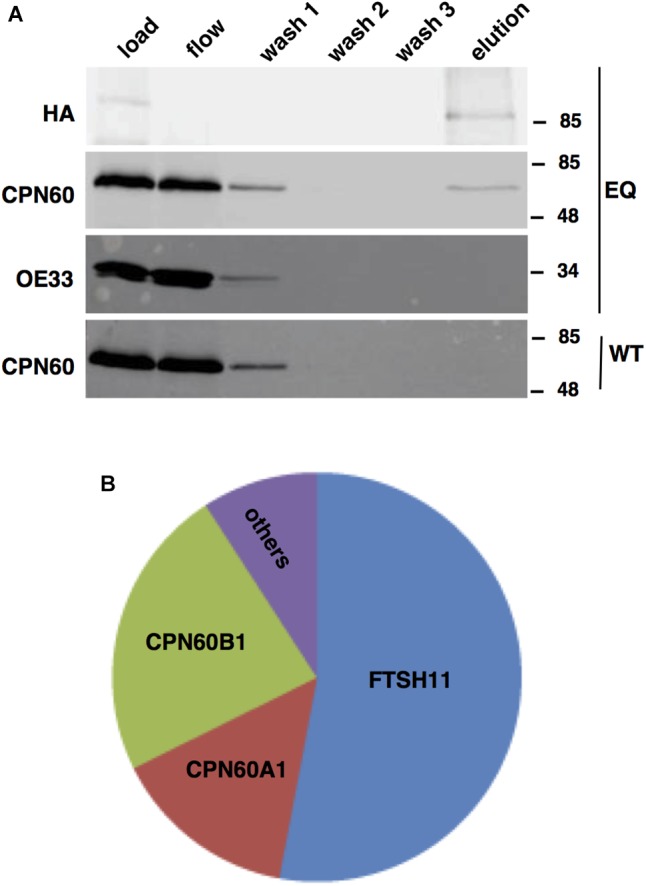
FTSH11-HA associates with CPN60. Total protein extracts from WT and EQ plants were solubilized with β-dodecyl maltoside, loaded on an immobilized anti-HA columns, thoroughly washed, and eluted with HA peptide. Samples from the different steps of the purification procedure were analyzed. **(A)** Immuno-blot analysis with HA, CPN60, and OE33 antibodies. Antibodies used are indicated on the left and migration of MW markers (in kDa) are indicated on the right of each blot. The genotypes of the samples are denoted on the far right of the blots. **(B)** Summary of MS analysis of the eluted fraction. The area of each section in the chart represents the abundance of the identified proteins within the eluted fraction from the EQ plants.

Unlike bacterial FTSH hexamers, which are products of single genes, the thylakoid FTSH hexameric complex comprises of four different subunits, FTSH1, FTSH2, FTSH5, and FTSH8 (Zaltsman et al., 2005; Sakamoto, 2006; Moldavski et al., 2012). This, together with the results of proteomic analyses of chloroplast envelope in which other FTSH proteins were found, raised the possibility that FTSH11 interacts with other FTSH subunits in the envelope. To test this hypothesis, FTSH11 was immuno-precipitated with a HA antibody from the EQ transgenic plants. To increase the stringency of this assay, we used total leaf extracts and washed the precipitates thoroughly prior to elution of the bound proteins with excess of HA peptides. As shown in Figure 3A, all of the HA-tagged protein was adsorbed to the matrix, as no signal appeared either in the flow-through or the different washes. Only upon washing the matrix with free HA peptide (“elution” in Figure 3A) the HA-tagged protein was released. To test whether other FTSHs, or any other proteins interacted with FTSH11, the eluted material was subjected to MS analysis. As expected, more than 50% of the protein material associated with the anti-HA matrix was identified as FTSH11 (Figure 3B). The two other proteins highly represented in the eluted material were CPN60B1 and CPN60A1. Less than 10% of the eluted material was identified as different chloroplast proteins. These same proteins were identified in control immuno-precipitations, performed on either protein extracts from WT plants or transgenic plants expressing HA-tagged rhomboid protease (Knopf et al., 2012). Thus, these proteins were considered as contaminants rather than substrates or components of the FTSH11 complex. Remarkably, unlike the thylakoid FTSH complex, FTSH11 was not associated with any other FTSH protein. Neither FTSH7, FTSH9, FTSH12, nor FTSHi1-4, all residing in the chloroplast envelope, were identified in the immuno-precipitated material.

In light of the results of the MS analysis, the fractions of the immuno-precipitation experiment were further subjected to immuno-blot analysis (Figure 3A). The eluted fraction, containing FTSH11, reacted also with a CPN60 antibody, but not with an antibody against the abundant chloroplast protein OE33 of the oxygen-evolving complex. In the mock immuno-precipitation from WT plants, no CPN60 was precipitated, suggesting that the interaction between CPN60 and FTSH11 was indeed specific. The intensity of the CPN60 band in the flow and first wash fractions, compared with that of the load, suggests that only a small fraction of the chloroplast CPN60 associates with FTSH11 (Figure 3A).

**FIGURE 4:**
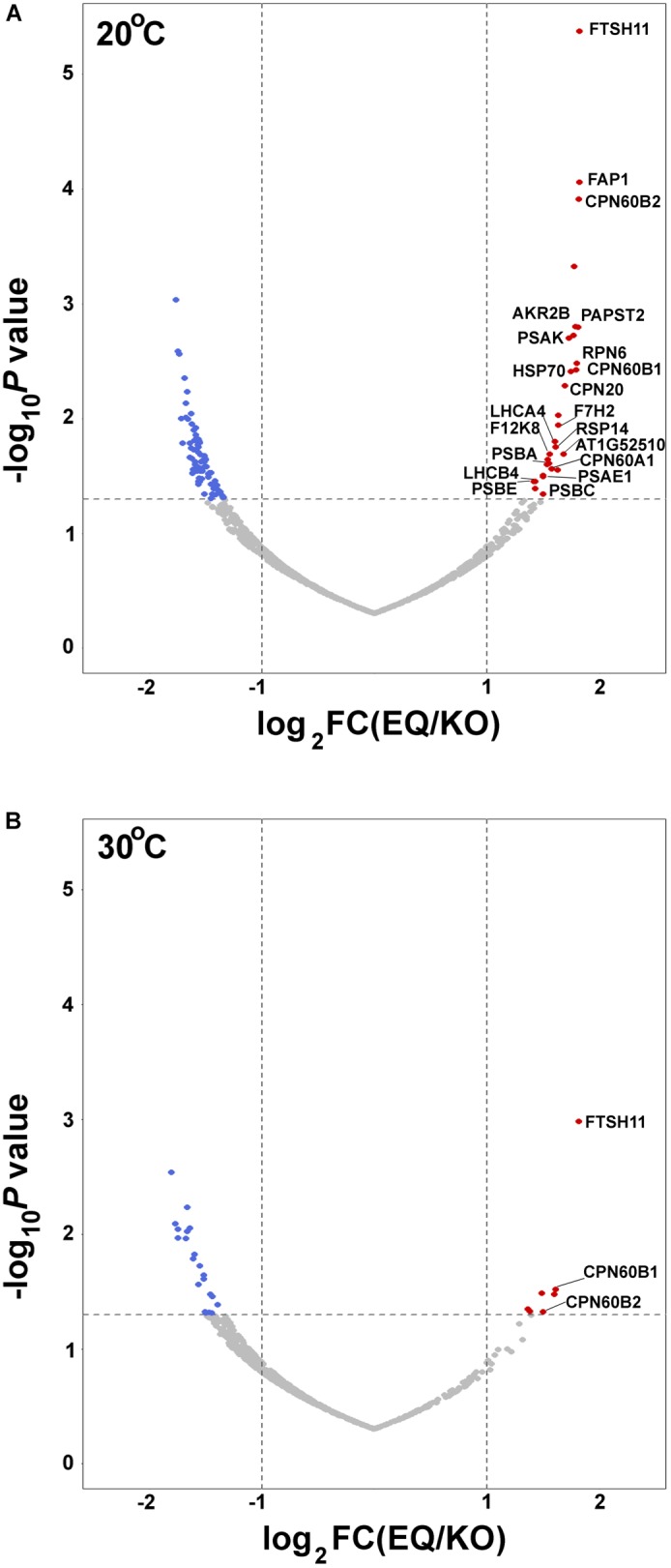
Affinity enrichment of FTSH11-HA. Total protein extracts from EQ and KO plants were solubilized with β-dodecyl maltoside, incubated with immobilized anti-HA beads, and slightly washed. Proteins associated with the beads were then subjected to MS analysis. Volcano plots are presented. Significantly enriched chloroplast proteins interacting with FTSH11 are depicted in red. (De-enriched proteins are in blue, and proteins that were not significantly changed are in gray.) **(A)** Samples from plants grown at 20°C. **(B)** Samples from plants grown at 20°C and then exposed to 30°C for 6 h.

To further unravel interactions between FtsH11 and potential interactors or substrates, we used the affinity enrichment approach (Keilhauer et al., 2015). Here the immuno-precipitated material, from either the HA-tagged line (EQ) or the reference KO one, is only slightly washed, the proteins associated with the HA beads are not eluted, and the material is subjected to MS analysis, followed by statistical analysis to distinguish true interacting proteins from background ones (Supplementary Table 1). The volcano plot presented in Figure 4A shows, as expected, strong enrichment of FTSH11 in the material precipitated from plants grown at 20°C. Interestingly, none of the other FTSH envelope proteins is found enriched. In agreement with the results of the affinity purification presented in Figure 3, CPN60 proteins are also enriched, including CPN60A1, CPN60B1, and CPN60B2, as well as their co-chaperone CPN20.

In addition to CPN60 proteins, other chloroplast proteins were found enriched along with the FTSH11 bait. One protein that co-localizes with FTSH11 is the nucleotide antiporter PAPST2 (Ashykhmina et al., 2019) (also designated EAAC). Proteins residing in the stroma, found associated with FTSH11 include the fatty acid binding protein FAP1, the chaperone HSP70, a hydrolase of unknown function (At1g52510), the ribosomal protein RPS14, the aldolase F12K8 and the glyceraldehyde-3-phosphate dehydrogenase GAPC2 (Figure 4A). Thylakoid proteins were also identified among the FTSH11-interacting proteins, including the PSI reaction center proteins PSAE1, PSAK, and their antenna protein LHCA4, the PSII reaction center proteins PSBA, PSBC and PSBE, and their antenna protein LHCB4. As the bait protein was proteolytically inactive, all these might be considered as potential trapped substrates.

This experiment was repeated with the same plants, but prior to the affinity enrichment step, the plants were exposed to 30°C for 6 h. For a reason that is not obvious to us, the only proteins enriched, in addition to FTSH11 itself, were CPN60B1 and CPN60B2 (Figure 4B), further highlighting the interaction between CPN60s and FTSH11.

### Comparative Proteomics – WT vs. KO, at 20 and 30°C

**FIGURE 5:**
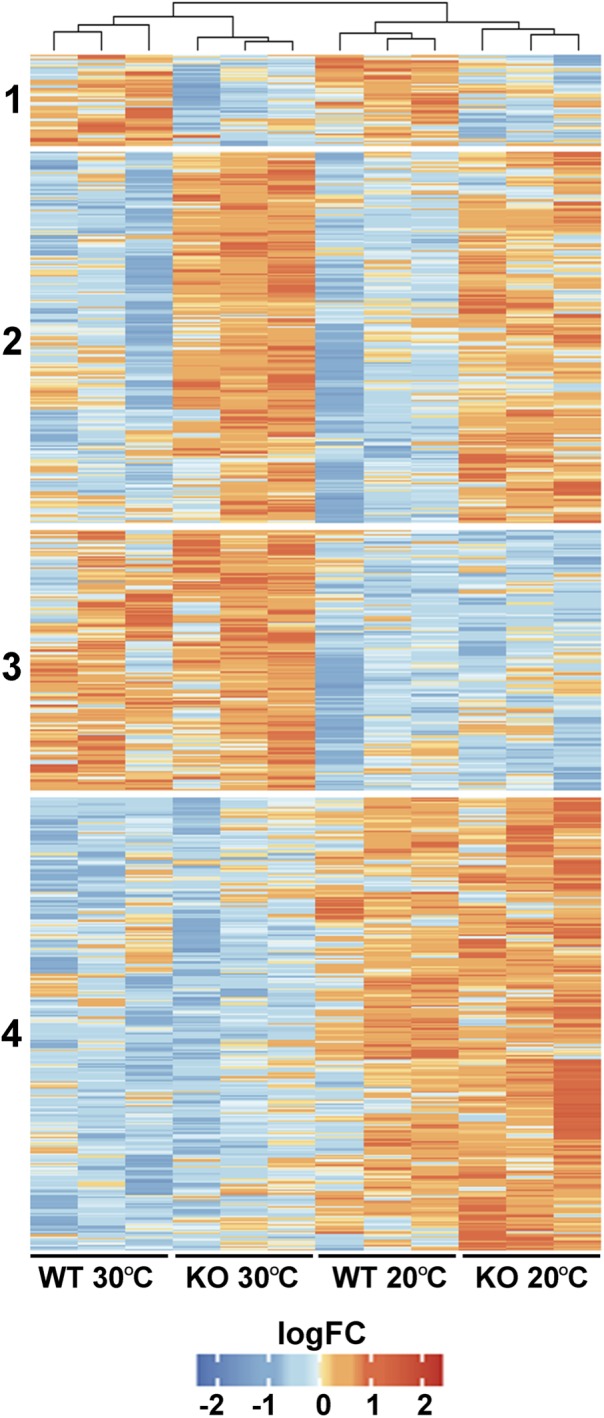
Comparative proteomic analysis of WT and FTSH11 KO mutant. WT and mutant plants were grown at 20°C for 4 weeks and then either exposed or not to 30°C for 6 h. Total protein was extracted from leaves and subjected to label-free quantitative LC-MS/MS analysis. Relative levels of all differentially expressed proteins in the two genotypes and two temperature treatments and their clustering are presented.

To gain an insight into the physiological role of FTSH11, a comparative proteomics approach was used on WT and FTSH11 KO plants. Plants were grown for 4 weeks at optimal temperature (20°C) and then remained at the same temperature or exposed to elevated temperatures (30°C) for 6 h. This length of exposure was chosen in order to unravel initial response to heat, before any visual symptoms can be detected. Of the 4,591 proteins identified by LC-MS/MS (Supplementary Table 2), 3,390 were identified by more than one peptide, allowing their reliable quantification. Of these, 708 proteins were differentially accumulated in at least one of the comparisons (fold change >2, *p*-value <0.05). As shown in Figure 5, four main characteristic patterns of accumulation could be observed: (1) Proteins downregulated in the KO mutant, regardless of the temperature treatment; (2) Proteins upregulated in the KO regardless of temperature; (3) Proteins upregulated at 30°C, irrespective of the genotype; and (4) Proteins downregulated at 30°C in both genotypes.

**FIGURE 6:**
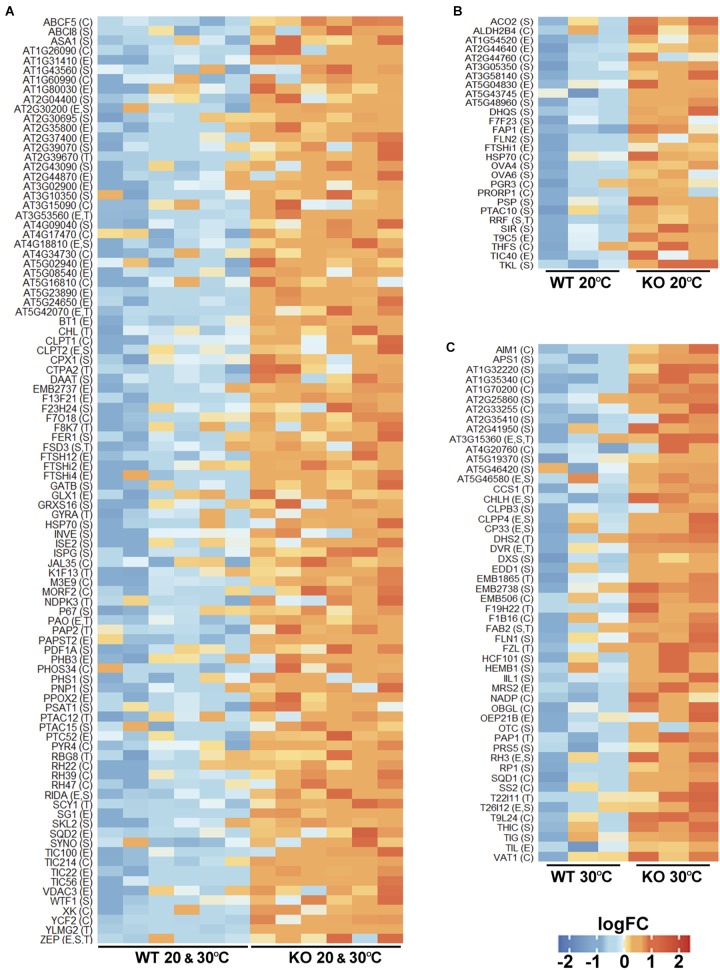
Chloroplast proteins upregulated in the FTSH11 KO mutant. Chloroplasts upregulated proteins were selected from the differentially expressed proteins presented in Figure 5. **(A)** Proteins that were upregulated both at 20°C and following exposure to 30°C for 6 h. **(B)** Proteins that were upregulated only at 20°C. **(C)** Proteins upregulated in the mutant only after exposure to 30°C. Protein names are denoted on the left of each panel. Intra-organelle localizations are in parentheses. C, chloroplast; E, envelope; S, stroma; T, thylakoid.

As upregulation of proteins in the protease KO mutant may result from their reduced turnover, such proteins can be candidate FTSH11 substrates. We thus took a closer look at all these proteins. Most of them were upregulated in the KO at both temperatures (Figure 6A). Noteworthy in this category were four components of the YCF2 inner envelope complex, which serves as a protein import motor (Kikuchi et al., 2018). These included FTSH12, a close homolog of FTSH11, the proteolytically inactive homologs FTSHi2 and FTSHi4, and YCF2 itself. Another component of the YCF2 complex, FTSHi1, accumulated to higher level in the mutant only at 20°C (Figure 6B). Moreover, four components of the Tic complex, through which proteins are being imported into the chloroplast (Kovacs-Bogdan et al., 2010; Kikuchi et al., 2013), are also upregulated: TIC214 (YCF1), TIC100, TIC56, and TIC22 (Figure 6A). Another protein implicated in precursor import, TIC40 is upregulated at 20°C (Figure 6B). Thus, it appears that FTSH11 is involved in regulating the level of the chloroplast import machinery at the inner envelope membrane.

Hints for impairment of the proteolytic capacity in the stroma upon the loss of FTSH11 protease can be seen in the upregulation of stromal chaperones such as two different HSP70s (Figure 6A,B) and CLPB3 (Figure 6C). Consistent with this is the over-accumulation in the mutant of one proteolytic component of the CLP protease (Olinares et al., 2011), CLPP4, and two assembly factors of this protease, CLPT1 and CLPT2 (Figure 6A).

Given the essentiality of FTSH11 to thermotolerance, it was interesting to see which chloroplast proteins were upregulated in the KO mutant upon exposure to elevated temperature, as these could be substrates of the protease. Among the numerous such proteins, several were noteworthy. First, two of the four aforementioned CLP proteins, CLPB3 and CLPP4, were found in this list (Figure 6C). Second, TIL, which was previously found to be required for thermotolerance (Chi et al., 2009), also over-accumulated in the absence of FTSH11. Consistent with the pale phenotype observed in the mutant at elevated temperatures (Figure 1), two protein involved in chlorophyll biosynthesis, CHLH and DVR are upregulated. Another interesting protein in this list is THIC. This stromal protein is involved in thiamine biosynthesis (Coquille et al., 2013), and was found to be one of the 20 most rapidly degrading proteins in Arabidopsis leaves (Li et al., 2017). All these proteins should be considered as candidate substrates of FTSH11 at elevated temperatures in future studies.

Most interesting among the upregulated proteins are three proteins that were found also trapped in the proteolytically inactive FTSH11 (Figure 4). These included PAPST2, the nucleotide antiporter located in the chloroplast envelope and mitochondria, the stromal chaperone HSP70, both over-accumulating in the KO mutant at both temperatures (Figure 6A), and the fatty acid binding protein FAP1 upregulated in the mutant at 20°C (Figure 6B). The identification of these three proteins by two independent experimental approaches strongly suggest that they are physiological substrates of the FTSH11 protease.

### Downregulation of TIC40 Upon Recovery From Heat Is Impaired in FTSH11 KO Mutant

**FIGURE 7:**
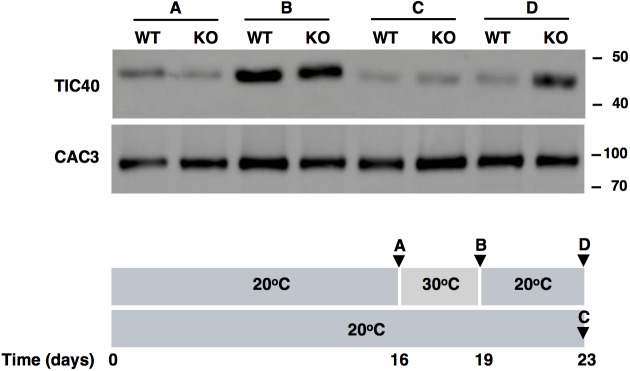
TIC40 is stabilized in the FTSH11 KO mutant upon recovery from heat. Two weeks old seedlings were grown at 20°C for additional 16 days, before being transferred to 30°C for 3 days and then returned to recovery at 20°C for 4 days. A control group was kept at 20°C for the entire duration of the experiment. At the time points indicated (**A–D** in the lower panel), samples were taken, total leaf extracts were made, and subjected to immunoblot analysis (upper panel) with the antibodies indicated in the left of the panel. Antibodies used are indicated on the left and migration of MW markers (in kDa) are indicated on the right of each blot.

As the level of components of the TIC complex appear to regulated by FTSH11 (Figure 6A,B), we tested the level of TIC40 in WT and KO plants before and after exposure to high temperature, and following a recovery period at optimal one, by immunoblot analysis. As can be seen in Figure 7, after 3 days at 30°C, the level of TIC40 in both WT and KO plants is highly elevated, consistent with the results of the MS analysis after a short exposure to heat (Figure 6B). In contrast, the level of CAC3, an unrelated envelope protein, remains quite constant. After transferring the WT plants back to 20°C, the level of TIC40 drops back to its original level whereas the level in the KO plants remains elevated. These results support the notion that the level TIC40 is regulated by FTSH11, probably by proteolytic degradation of excess copies of the protein.

## Discussion

When it comes to determining the physiological role of a given FTSH protease, the immediate question that comes to mind is whether this role is dependent on the proteolytic or the ATPase activity of the protease. The relevance of this question is reinforced due to the evolutionary history of FTSH proteins, during which a number of proteolytically inactive FTSHs (designated FTSHi) have been evolved. In the case of Arabidopsis, there are five such proteins, all of them are essential for development and growth (Sokolenko et al., 2002; Wagner et al., 2012; Mishra et al., 2019). Four of these have been recently identified in a large chloroplast complex, together with FTSH12, serving as a motor for protein import into chloroplasts (Kikuchi et al., 2018). Using a complementation assay with proteolytically active and inactive proteins, we found here that the proteolytic activity of FTSH11 is essential for conferring resistance to elevated temperatures (Figure 1). This finding is in contrast to the case of FTSH12, whose proteolytic activity is dispensable for its activity in protein import (Kikuchi et al., 2018).

The localization of most if not all of FTSH11 to the chloroplast inner envelope membrane, the same sub-compartment where FTSH12 and the five FTSHi proteins are located, raised the suspicion that it could be a component of a large heteromeric FTSH complex, similar to the one found in the thylakoid, which is composed of four different gene products (Zaltsman et al., 2005). However, we could not find any other FTSH protein associated with FTSH11 in either affinity purification or affinity enrichment assays (Figure 3, 4, respectively), suggesting that it forms a homo-complex. This conclusion is further supported by the aforementioned recent study (Kikuchi et al., 2018), where five envelope FTSH proteins were found in complex, but no FTSH11 was associated with them.

The most surprising finding of this study is probably the association of stromal CPN60 proteins with the FTSH11 complex. This association was observed in both affinity purification and affinity enrichment assays, when proteolytically inactive FTSH11 was used as a bait (Figure 3, 4, respectively). Moreover, CPN60s were associated with FTSH11 when the affinity enrichment assay was carried out after exposure to high temperature as well (Figure 4B), where only a few proteins could be identified along with FTSH11. It is also interesting to note that when proteolytically active FTSH11 was used as a bait, we could not observe this interaction (unpublished observation). The functional and physiological significance of the interaction is not clear to us yet. However, it is interesting to note that when similar experiments were carried out on proteolytically inactive FTSH2, located in the thylakoid membrane, whose functional domains are also exposed to the stroma, no CPN60 was associated with it (unpublished observation), so the interaction is likely to be specific to FTSH11. These observations make it tempting to hypothesize that stromal substrates are recruited by CPN60 and delivered to FTSH11 for degradation. The interaction between the two complexes can be observed only when proteolytic degradation is inhibited, in this case, by a mutation in the proteolytic active site. This delivery hypothesis will have to be rigorously tested in the future.

To unravel potential substrates of FTSH11, two independent large-scale approaches were used. The less selective one was comparative proteomics, in which proteomes of total leaf extracts from two genotypes – WT and FTSH11 KO, at optimal and elevated temperatures were compared (Figure 5, 6). Here, potential substrates are expected to be upregulated in the mutant background. Nevertheless, over-accumulation of a given protein in the mutant can represent a pleotropic effect, thus other criteria, such as co-localization, need to applied. A more direct approach is substrate trapping, which is usually done with proteolytically inactive variants, allowing the binding of substrates to the protease, but in the absence of their processing, they can be identified along with the protease trap. Three proteins were identified in both assays: the nucleotide antiporter PAPST2, a stromal HSP70 involved in protein import (Su and Li, 2010), and the envelope fatty acid binding protein FAP1 (Ngaki et al., 2012). The physical proximity between FTSH11 and these proteins, being located in the envelope membrane or the stroma, together with their identification in two different assays, strongly suggest that these three proteins are indeed substrates of the FTSH11 protease who regulates their physiological levels.

Another interesting group of trapped proteins are integral thylakoid membrane proteins of PSI and PSII, seven of them in total (Figure 4A). None of these is found among the proteins upregulated in the mutant and they are located in a different membrane than FTSH11. Yet, as thylakoids fill up most of the chloroplast volume, contact between the thylakoid and the envelope membranes is not that scarce. Thus, it could be that an envelope protease participates in degradation of thylakoid membrane proteins. This possibility will have to be tested more directly in future experiments. Similarly, the plethora of envelope and stroma proteins that were found upregulated in the KO mutant, including the rapidly turned over THIC (Li et al., 2017), will have to be further examined as potential substrates of FTSH11.

Using a more targeted approach, we tested the level of a single protein that was found upregulated in FTSH11 KO plants, TIC40 (Figure 6), as a representative of the group of TIC proteins that demonstrated a similar behavior. The level of this protein increased at elevated temperatures in both the WT and KO plants (Figure 7), and decreased back to normal only in the WT but not in the KO upon return to normal temperature. This observation suggests that FTSH11, probably by virtue of its proteolytic activity, is responsible for degrading excess copies of TIC40, and probably other TIC components that were found upregulated in the mutant upon exposure to high temperature. This approach will be useful in future attempts to identify and characterize potential substrates of the FTSH11 protease.

## Materials and Methods

### Plant Material

Wild type (WT) and mutant *Arabidopsis thaliana* (ecotype Columbia) plants were grown under controlled conditions of 16 h-light/8 h-dark cycles, with a photon flux density of ∼100 μmol m^−2^ s^−1^, at 20°C for 4 weeks on moistened Kekkila peat. When needed, plants were transferred to 30°C for additional 6 h. Alternatively, surface sterilized seeds were sown on sterile × 0.5 MS media with 1% sucrose and B5 vitamins, stratified for 2 days at 4°C in the dark, and then grown under long-day conditions for 16 days at either 22, 27 or 30°C.

The FTSH11 knockout (KO) *salk033047* was complemented with either a full length FTSH11 cDNA, encoding also for 3xHA tag at the C-terminus of the protein (EE line), or a similar construct where residues H620 and E621 in the proteolytic active site were replaced by Q (EQ line). The HA tagging and the site-directed mutagenesis were done as previously described (Adam, 1995; Moldavski et al., 2012), respectively. Both constructs contained upstream of the coding region a 2-kb sequence of the FTSH11 native promoter.

### Organelle Isolation and Fractionation

Intact chloroplasts and mitochondria were isolated by density gradient centrifugation on Percoll gradients as described in Halperin et al. (2001). Chloroplasts were further fractionated by osmotic shock followed by sonication and centrifugation on sucrose gradients as described (Knopf et al., 2012).

### Protein Extraction, Immunoblot Analysis, Affinity Purification and Affinity Enrichment, and MS Analysis

Detailed descriptions of protein extraction, immunoblot analysis and affinity enrichment were recently published (Butenko et al., 2018). The antibodies used were the following: HA (Abcam), CPN60 and TIC40 (from Agrisera), OEP24 and CAC3 from the J. Soll and E. Wurtele labs, respectively. OE33 and ClpX antibodies were described in Itzhaki et al. (1998) and Halperin et al. (2001), respectively. In principle, denatured protein extracts included 8% (w/v) SDS and 5 M urea, whereas non-denatured extracts were solubilized by 1.0% (w/v) n-dodecyl-β-D-maltoside (DDM, Tivan-Biotech). All extractions were done in the presence of Protease Inhibitor Cocktail for plant (Sigma-Aldrich). Both affinity purification and enrichment were done with anti-HA Agarose seven beads (Abcam). In the affinity purification experiments, the bound proteins were eluted by incubating the washed beads with excess of HA peptide, whereas in the affinity enrichment experiments the washed beads were further analyzed as is (thus containing specifically as well as non-specifically bound proteins). All MS analyses were also performed as recently described (Butenko et al., 2018).

### Data Processing, Statistical and Bioinformatic Analyses

Mass spectrometry individual intensities were log_2_ transformed and *Z*-score normalized. Missing values were imputed using normal distribution, with a mean and standard deviation adjusted to resemble low-abundant proteins signals. Proteins were considered for comparative analysis if a protein was identified in at least two out of three replicates and by a minimum of two peptides. Two-way analysis of variance (ANOVA) was used to identify significant differences across the biological replicates. The criteria used to denote significantly, differentially expressed proteins for further analysis were fold-change >2 and *p*-value <0.05. The quality of the expressed and differentially expressed data was examined by principal component analysis (PCA). Heat maps were based on K-means clustering, using Pearson correlation coefficient as a distance metric. The optimal number of clusters was computed using the gap statistic method and comprised of 1000 Monte Carlo iterations (Tibshirani et al., 2001). Volcano plots of the CoIP-MS signals were constructed following log_2_ transformation, *Z*-score normalization and a pairwise Student’s *t*-test (*p*-value <0.05). Proteins were annotated using Blast2Go software (Conesa and Gotz, 2008), the AT_CHLORO (Bruley et al., 2012), or PPDB (Sun et al., 2009) databases. Analyses were performed using R/Bioconductor (version R.3.4.2).

## Data Availability

All datasets for this study are included in the manuscript and the Supplementary Files.

## Author Contributions

ZA and JC conceived the project and designed the experiments. AK-P, MR, and LN performed the different experiments. AS did all manuscript analyses. EA-S and MR processed all data and did the statistical and bioinformatics analyses. ZA wrote the manuscript and all co-authors read and approved it.

## Conflict of Interest Statement

The authors declare that the research was conducted in the absence of any commercial or financial relationships that could be construed as a potential conflict of interest.
